# Iron deficiency in primary care patients with heart failure: a cross-sectional study of the heart failure in Southern Sweden (HISS) cohort

**DOI:** 10.1186/s12872-026-06207-8

**Published:** 2026-07-03

**Authors:** Fredrik Vinge, Oscar Braun, Moa Wolff, J. Gustav Smith, Kristina Sundquist, Veronica Milos Nymberg

**Affiliations:** 1 Center for Primary Health Care Research, Department of Clinical Sciences Malmö, Lund University, Jan Waldenströms gata 35, Malmö, 214 28 Sweden; 2 University Clinic Primary Care, Skåne University Hospital, Malmö, Region Skåne Sweden; 3 Department of Cardiology, Department of Clinical Sciences Lund, Lund University and Skåne University Hospital, Lund, Sweden; 4 Department of Molecular and Clinical Medicine, Institute of Medicine, University of Gothenburg, SciLifeLab and Sahlgrenska University Hospital, Gothenburg, Sweden; 5 Wallenberg Centre for Molecular Medicine and Lund University Diabetes Centre, Lund University, Lund, Sweden

**Keywords:** Iron deficiency, Heart failure, Primary care, Symptom burden, Prevalence

## Abstract

**Background:**

Iron deficiency is a common and clinically relevant comorbidity in heart failure, associated with reduced functional capacity, higher symptom burden, and increased hospitalisation risk. Most evidence on iron deficiency in heart failure originates from hospital-based cohorts, whereas the epidemiology and clinical implications in primary care populations remain poorly described. This study aimed to determine the prevalence of iron deficiency among primary care patients with heart failure in southern Sweden and to examine its association with symptom severity.

**Methods:**

This cross-sectional analysis used baseline data from the Heart Failure in Southern Sweden study, a prospective intervention project conducted at 20 primary health care centres. Adult patients with heart failure across all left ventricular ejection fraction categories were included. Iron deficiency was defined as transferrin saturation < 20%.

**Results:**

In total, 466 primary care patients with heart failure were included, of whom 124 (26.7%) had iron deficiency. Symptom severity was higher in patients with iron deficiency: 35.5% were classified as New York Heart Association (NYHA) class III–IV, compared with 18.7% among patients without iron deficiency. Similar findings were observed in the subgroup with left ventricular ejection fraction below 50%, where 25.2% had iron deficiency and 42.6% were classified as NYHA III–IV compared with 18.4% among patients without iron deficiency. In multivariable analysis adjusting for clinically relevant covariates, iron deficiency remained associated with NYHA class III–IV (OR 1.97, 95% CI 1.18–3.31, *p* = 0.010).

**Conclusions:**

Iron deficiency is common among patients with heart failure managed in primary care and remained associated with higher symptom burden after adjustment for prespecified covariates. These findings highlight the potential clinical relevance of assessment of iron status in primary care.

**Trial registration:**

ClinicalTrials.gov, NCT04129658. Registered on 15 October 2019.

**Supplementary Information:**

The online version contains supplementary material available at 10.1186/s12872-026-06207-8.

## Background

Heart failure is a major global health challenge, affecting approximately 64 million people worldwide. Its prevalence among adults is estimated at 1–3% and continues to rise, driven by aging populations and improved survival after myocardial infarction [1, 2]. Despite therapeutic advances, heart failure remains associated with high morbidity and mortality, with frequent hospitalisations and one-year case-fatality rates of 24–33% [3].

Iron deficiency is a common comorbidity in heart failure, affecting approximately 40% of patients, and its prevalence increases with worsening heart failure symptoms according to the New York Heart Association (NYHA) classification [4], where class I represents no physical activity limitations and class IV represents symptoms at rest. Observational studies and randomized trials consistently demonstrate that iron deficiency is associated with greater symptom burden, reduced exercise capacity, and increased risk of hospitalisation [5–7]. The European Society of Cardiology recommends intravenous iron for patients with symptomatic heart failure with a left ventricular ejection fraction (LVEF) < 50% and iron deficiency to improve symptoms, quality of life and reduce the risk of heart failure hospitalisation [8–12]. In contrast, these benefits have not been demonstrated with oral iron supplementation [13].

Functional iron deficiency, characterized by inadequate intracellular iron despite normal or elevated ferritin levels due to inflammation, is common in patients with heart failure. Traditional guideline criteria therefore define iron deficiency as ferritin < 100 µg/L, or ferritin 100–299 µg/L with a transferrin saturation (TSAT) < 20% [6, 14–16]. Using this definition, approximately 50% of patients with heart failure in specialised clinics meet criteria for iron deficiency [17].

However, ferritin is an acute-phase reactant and may be falsely elevated in the presence of chronic inflammation, potentially masking true iron depletion. As TSAT < 20% better reflects the amount of circulating iron available for cellular uptake and correlates more closely with bone marrow iron stores, several recent studies have focused on defining iron deficiency solely by TSAT [18–20]. A TSAT-based definition more precisely identifies patients who benefit from intravenous iron therapy and may provide a more accurate assessment of clinically relevant iron deficiency in heart failure [10, 11, 20]. Consequently, a TSAT-based definition is increasingly favoured over combined ferritin and TSAT criteria in heart failure. Previous studies have suggested, however, that prevalence estimates may not differ substantially between definitions, although findings have been inconsistent [6, 16, 21]. However, most of these studies have been conducted in hospital settings or specialised outpatient clinics, often involving patients with advanced heart failure or recent hospitalisations [22, 23]. This raises concerns about the generalizability of these findings to primary care populations, where patients with heart failure tend to be older, have more comorbidities, and present with milder symptoms.

Primary care in Sweden manages a large proportion of patients with chronic heart failure [24, 25]. Evidence on the prevalence of iron deficiency and its association with symptom severity in primary care-managed patients with heart failure is, to the best of our knowledge, lacking. This represents a critical knowledge gap, as early identification and treatment of iron deficiency in primary care-managed heart failure could improve symptoms and reduce hospitalisations.

The aim of this study was to determine the prevalence of iron deficiency among patients with heart failure in a Swedish cohort of primary care patients and to compare the severity of heart failure symptoms (NYHA class) between patients with and without iron deficiency.

## Methods

### Study design

The Heart Failure in Southern Sweden (HISS) project was a prospective intervention study conducted in primary care to assess adherence to guideline-directed medical therapy (GDMT) and to evaluate the effectiveness of a case-based, cardiologist-supervised educational program aimed at improving adherence to GDMT. The present study was conducted using data collected at baseline within the project.

### Study setting

Between 2020 and 2023, all 166 primary health care centres (PHCCs) in the southernmost region of Sweden (Region of Skåne, with a population of approximately 1.4 million inhabitants) were invited to participate in the HISS study. Primary care in Sweden is tax-funded, and patients are listed at a centre of their choice, which provides first-line management of chronic conditions, including heart failure. A total of 20 PHCCs, including both public and private institutions serving urban and rural populations, participated in the study [26].

### Study participants

Adult patients (≥ 18 years) with a diagnosis of heart failure (ICD-10 codes I50, I11.0, I42, I43) recorded in their medical records were eligible for inclusion in the HISS study. At each participating PHCC, a research assistant identified patients with heart failure and sent invitation letters to assess their interest in participation. Participating physicians subsequently reviewed the clinical information collected in the case report forms and had the opportunity to reassess the heart failure diagnosis. Patients with hypertrophic cardiomyopathy (ICD-10 codes I42.1 and I42.2) and those receiving municipal home care services were excluded, as the HISS cohort was designed to include patients able to provide informed consent and to participate in the study procedures at the PHCC. A total of 587 patients were recruited to the HISS study. Of these, 466 patients with complete data on NYHA class and iron status were included in the present analysis.

### Data collection and assessment

Data regarding echocardiographic findings and NYHA functional class were extracted from the electronic medical records and recorded in case report forms by a designated nurse or physician at each participating primary health care centre. At the baseline visit, blood samples were obtained and anthropometric measurements (weight and height) were collected. Baseline variables included age, sex, body mass index (BMI), NYHA functional class, LVEF, and laboratory measurements including haemoglobin, ferritin, transferrin saturation (TSAT), serum iron, total iron-binding capacity (TIBC), and NT-proBNP. NYHA class was obtained from routine clinical assessments documented in the electronic medical records and recorded in the case report forms by a designated nurse or physician at each participating primary health care centre at the study visit. No central adjudication or standardisation procedure was performed across centres.

NT-proBNP was log-transformed due to its skewed distribution. Transferrin saturation (TSAT) was calculated as serum iron divided by total iron-binding capacity. Iron deficiency was defined as TSAT < 20%. This represents a TSAT-based definition and differs from the conventional ESC guideline definition, which defines iron deficiency as ferritin < 100 µg/L or ferritin 100–299 µg/L together with TSAT < 20%. We used this definition because ferritin is an acute-phase reactant and may be elevated in the presence of chronic inflammation, potentially masking iron deficiency. TSAT has therefore been suggested to provide a more accurate assessment of biologically relevant iron deficiency in patients with inflammatory conditions, including heart failure.

### Statistical analysis

Continuous variables are presented as mean ± standard deviation (SD) or median with interquartile range (IQR), as appropriate, and categorical variables as numbers and percentages. Group differences between patients with and without iron deficiency were analysed using the Pearson χ^2^ test for categorical variables and independent samples t-test or Mann–Whitney U test for continuous variables, depending on data distribution. The association between iron deficiency and NYHA functional class was assessed using the Pearson χ^2^ test.

For the main analyses, all patients with available data on TSAT and NYHA class were included, irrespective of LVEF. In a subgroup analysis restricted to patients with reduced or mildly reduced LVEF, patients with missing ejection fraction data were excluded (*n* = 7).

To further examine the association between iron deficiency and symptom severity, multivariable logistic regression analysis was performed with NYHA class III–IV as the dependent variable. The model was adjusted for age, sex, body mass index, haemoglobin, NT-proBNP and ejection fraction phenotype. These variables were selected based on their established associations with heart failure severity and their potential relationships with iron deficiency. Sensitivity analyses were performed by excluding patients with anaemia, by restricting the analysis to patients with LVEF < 50%, and by applying the ESC guideline definition of iron deficiency. Agreement between the TSAT-based and ESC guideline-based definitions of iron deficiency was assessed using Cohen’s kappa statistic. Additional sensitivity analyses were performed with further adjustment for SGLT2 inhibitor and loop diuretic treatment, as these therapies differed significantly between patients with and without iron deficiency.

A two-sided p-value < 0.05 was considered statistically significant. Statistical analyses were performed using IBM SPSS Statistics, version 30 (IBM Corp., Armonk, NY, USA). ChatGPT (OpenAI) was used for language editing assistance. The authors reviewed all content and take full responsibility for the manuscript.

### Ethical considerations

The study was conducted in accordance with the principles of the Declaration of Helsinki. Written informed consent was obtained from all participants prior to inclusion. Ethical approval was granted by the Swedish Ethical Review Authority (Dnr 2019–03944).

## Results

Of the 587 participants initially enrolled, 121 were excluded due to missing NYHA class and/or TSAT (117 missing NYHA class and 6 missing TSAT; some had both missing). Characteristics of the excluded patients are presented in Supplementary Table S1. Included and excluded patients were broadly similar with respect to age, sex, iron status, haemoglobin concentration and prevalence of anaemia. Included patients had slightly higher NT-proBNP concentrations. Among these, 7 also had missing values for LVEF and were therefore not available for the subgroup analysis of patients with LVEF < 50%. This resulted in a final study population of 466 patients with heart failure, of whom 124 (26.7%) had iron deficiency according to the TSAT-based definition. Using the ESC guideline definition, 293 patients (62.9%) fulfilled criteria for iron deficiency. Thus, the estimated prevalence differed substantially depending on the definition used. Agreement between the two definitions was low (Cohen’s κ = 0.20; Supplementary Table S2). Median age was 79 years and was similar between groups. Likewise, body mass index (BMI) was comparable between patients with and without iron deficiency. Patients with iron deficiency (TSAT < 20%) had higher NT-proBNP concentrations (median 1365 vs. 1014 ng/L, *p* = 0.009) and lower haemoglobin values (mean 131.5 vs. 140.3 g/L, *p* < 0.001). Overall, 41% of the cohort were women. Iron deficiency was present in 29.3% of women and 24.8% of men, with no statistically significant difference between sexes (*p* = 0.280). Anaemia was present in 19.6% of the cohort and was more common in patients with iron deficiency than in those without (30.9% vs. 15.5%, *p* < 0.001); however, most patients with iron deficiency did not have anaemia (Table 1). Anaemia was significantly associated with higher NYHA class, with a greater proportion of anaemic patients in NYHA III–IV compared with non-anaemic patients (33.3% vs. 20.4%, *p* = 0.006).

**Table 1:** Baseline characteristics of patients with and without iron deficiency

Variable	All (*n* = 466)	ID (*n* = 124, 26.7%)	Non-ID (*n* = 342, 73.3%)	*p*-value
Age (years)	79 (73–84)	80 (75–84)	79 (73–83)	0.123
BMI (kg/m^2^)	27.5 (25.9–31.9)	27.5 (24.7–33.4)	27.5 (24.9–31.6)	0.649
Ferritin (µg/L)	84 (46–165)	46 (26–83)	100 (59–187)	< 0.001
Transferrin saturation (%)	25 (19–32)	15 (12–18)	28 (24–34)	< 0.001
NT-proBNP (ng/L)	1073 (412–2238)	1365 (542–2925)	1014 (397–1965)	0.009
Haemoglobin (g/L)	138.0 (16.1)	131.5 (16.3)	140.3 (15.4)	< 0.001
Women, n (%)	191 (41.0)	56 (45.2)	135 (39.5)	0.270
Anaemia, n (%)	91 (19.6)	38 (30.9)	53 (15.5)	< 0.001

Values are presented as median (IQR) unless otherwise specified. Haemoglobin is presented as mean (SD). Anaemia data missing for one participant

Iron deficiency was defined as transferrin saturation (TSAT) < 20%

*Abbreviations*: *BMI* body mass index, *ID* iron deficiency, *NT-proBNP* N-terminal pro-B-type natriuretic peptide, *IQR* interquartile range, *SD* standard deviation

In the subgroup of patients with reduced or mildly reduced LVEF (< 50%, *n* = 270), 68 (25.2%) had iron deficiency. Baseline characteristics were broadly similar to the overall cohort. See Supplementary Table S5.

Baseline heart failure pharmacotherapy according to iron deficiency status is presented in Supplementary Table S3. Patients with iron deficiency were more frequently treated with SGLT2 inhibitors and loop diuretics.

**Fig. 1:**
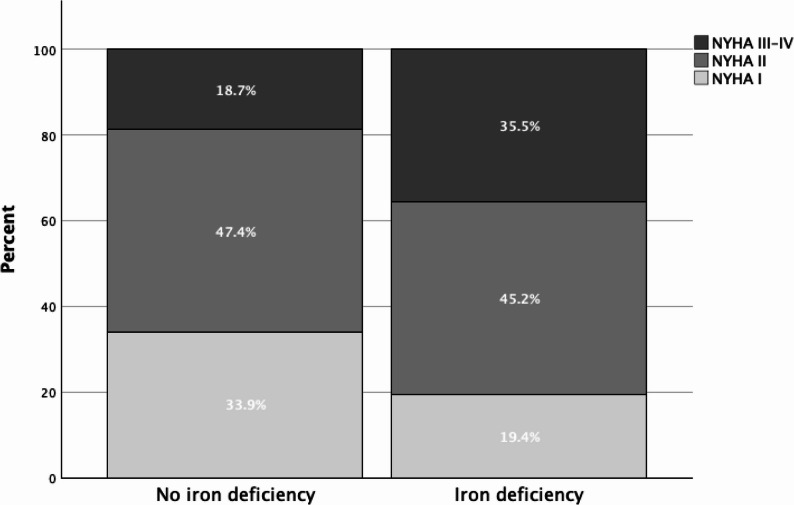
NYHA class in relation to iron status. Distribution of symptom severity according to New York Heart Association (NYHA) functional class in patients with and without iron deficiency (ID)(All EF, *n* = 466)

**Fig. 2:**
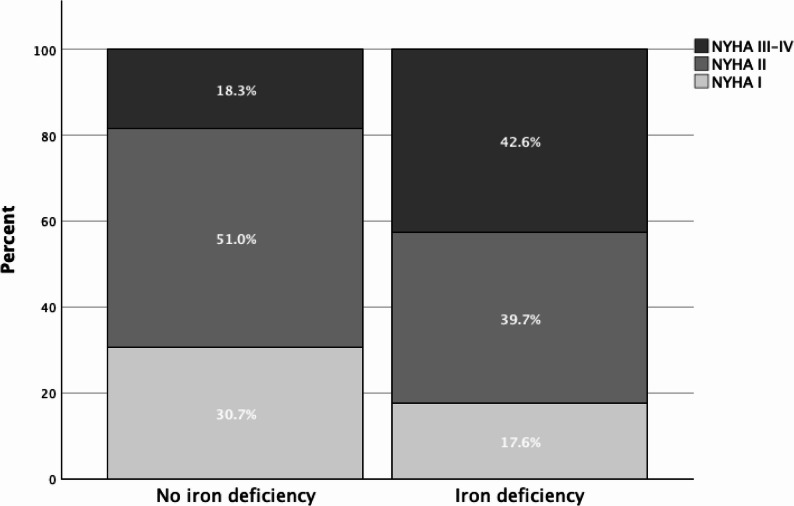
NYHA class in relation to iron status in patients with LVEF < 50%. Distribution of symptom severity according to New York Heart Association (NYHA) functional class in patients with and without iron deficiency (ID) among patients with left ventricular ejection fraction < 50% (*n* = 270)

Patients with iron deficiency had a higher symptom burden compared with those without iron deficiency. Specifically, 35.5% of patients with iron deficiency were classified as NYHA III–IV, compared with 18.7% of those without iron deficiency (*p* < 0.001, Table 2; Fig. 1). When patients with anaemia were excluded from the analysis, iron deficiency remained significantly associated with higher symptom burden (NYHA III–IV: 34.1% vs. 17.0%, *p* < 0.001). Similarly, in the subgroup with LVEF < 50% (*n* = 270), 42.6% of patients with iron deficiency were classified as NYHA III–IV compared with 18.4% of those without iron deficiency (*p* < 0.001, Table 3; Fig. 2).

**Table 2:** NYHA class by iron deficiency status

	All (*n* = 466)	ID (*n* = 124)	Non-ID (*n* = 342)
NYHA I	140 (30.0)	24 (19.4)	116 (33.9)
NYHA II	218 (46.8)	56 (45.2)	162 (47.4)
NYHA III–IV	108 (23.2)	44 (35.5)	64 (18.7)

Distribution of symptom severity according to New York Heart Association (NYHA) functional class in patients with and without iron deficiency. Values are presented as n (%). Group differences were analysed using the χ^2^ test (*p* < 0.001)

**Table 3:** NYHA class by iron deficiency status in patients with LVEF < 50%

	All (*n* = 270)	ID (*n* = 68)	Non-ID (*n* = 202)
NYHA I	74 (27.4)	12 (17.6)	62 (30.7)
NYHA II	130 (48.1)	27 (39.7)	103 (51.0)
NYHA III–IV	66 (24.4)	29 (42.6)	37 (18.3)

Distribution of symptom severity according to New York Heart Association (NYHA) functional class in patients with and without iron deficiency and left ventricular ejection fraction < 50%. Values are presented as n (%). Group differences were analysed using the χ^2^ test (*p* < 0.001)

In multivariable logistic regression adjusted for age, sex, BMI, haemoglobin, NT-proBNP and ejection fraction phenotype, iron deficiency defined as TSAT < 20% remained associated with higher symptom burden (NYHA III–IV) (OR 1.97, 95% CI 1.18–3.31, *p* = 0.010, Table 4). Additional adjustment for SGLT2 inhibitor and loop diuretic treatment, the only heart failure therapies that differed significantly between groups (Supplementary Table S3), did not materially alter the association (adjusted OR 1.87, 95% CI 1.11–3.14). In a sensitivity analysis excluding patients with anaemia, the association between iron deficiency and NYHA class III–IV was attenuated and no longer statistically significant (OR 1.32, 95% CI 0.59–2.94). In the subgroup of patients with LVEF < 50%, iron deficiency was not significantly associated with NYHA III–IV after multivariable adjustment (OR 1.35, 95% CI 0.54–3.37). Results of the sensitivity analysis using the ESC guideline definition are presented in Supplementary Table S4.

**Table 4:** Factors associated with NYHA class III–IV: logistic regression analysis

Variable	Crude OR (95% CI)	*p*-value	Adjusted OR (95% CI)	*p*-value
Iron deficiency	2.39 (1.51–3.77)	< 0.001	1.97 (1.18–3.31)	0.010
Age	1.03 (1.00–1.07)	0.038	1.00 (0.96–1.03)	0.841
Female sex	1.79 (1.16–2.76)	0.008	0.75 (0.46–1.23)	0.251
BMI (kg/m^2^)	1.04 (1.00–1.06)	0.055	1.07 (1.02–1.11)	0.003
Haemoglobin (g/L)	0.98 (0.96–0.99)	< 0.001	0.98 (0.97–1.00)	0.048
log NT-proBNP	1.72 (1.39–2.12)	< 0.001	1.66 (1.30–2.12)	< 0.001
LVEF < 50% vs. ≥ 50%	1.17 (0.75–1.83)	0.482	0.73 (0.44–1.20)	0.211

Crude and adjusted logistic regression analyses examining factors associated with higher symptom burden (NYHA III–IV). The multivariable model included 425 participants with complete data and comprised age, sex, body mass index, haemoglobin, log NT-proBNP, LVEF < 50% (vs. ≥ 50%) and iron deficiency. Odds ratios for continuous variables represent the change in odds per unit increase

*Abbreviations*: *BMI* body mass index, *CI* confidence interval, *NT-proBNP* N-terminal pro-B-type natriuretic peptide, *OR* odds ratio

## Discussion

The prevalence of iron deficiency in our cohort (27%) was lower than reported in several hospital-based heart failure cohorts [16]. Compared with hospital-based cohorts, our primary care patients were older and had a lower average NYHA class, indicating a lower heart failure symptom burden. Iron deficiency was associated with greater symptom severity, consistent with previous evidence linking iron deficiency to functional impairment in heart failure. Taken together, these findings support the clinical relevance of identifying iron deficiency in patients with heart failure in primary care, in relation to current ESC guideline recommendations [12].

A notable finding was the substantial difference in prevalence estimates depending on the definition used. While 26.7% of patients fulfilled the TSAT-based definition, 62.9% fulfilled the ESC guideline definition, and agreement between definitions was low. Although previous studies have reported differences between TSAT-based and ESC guideline-based definitions of iron deficiency, the discrepancy observed in the present cohort was more pronounced than that reported in hospital-based heart failure populations [6, 16]. This finding suggests that the impact of the chosen definition may differ between primary care and hospital-based heart failure populations. The reason for this remains uncertain but may relate to differences in patient characteristics, disease severity, comorbidity burden, or inflammatory status. Notably, a large proportion of patients in the present cohort had ferritin concentrations below 100 µg/L, which substantially increased the prevalence estimate when the ESC guideline definition was applied.

In multivariable analysis adjusting for several clinically relevant covariates, iron deficiency remained associated with higher symptom burden. However, the association was attenuated when patients with anaemia were excluded and in subgroup analyses restricted to patients with LVEF < 50%. These exploratory analyses involved substantially smaller sample sizes (*n* = 425 in the full model, *n* = 281 after exclusion of anaemia, *n* = 191 in the LVEF < 50% subgroup, and *n* = 165 when both anaemia and heart failure with preserved ejection fraction (HFpEF) were excluded), which reduced statistical power, limiting the ability to detect statistically significant associations. Negative findings in subgroup analyses should be interpreted cautiously due to limited statistical power. Iron deficiency and anaemia are closely related conditions, and haemoglobin levels may partly mediate the relationship between iron deficiency and symptom burden, making it difficult to fully disentangle their individual contributions.

Although iron deficiency was common, only a small proportion of patients fulfilled guideline-based criteria for intravenous iron therapy. In our cohort, only 56 patients (12%) had LVEF < 50%, iron deficiency and symptoms (NYHA ≥ II), and were therefore eligible for treatment. These findings support the feasibility of guideline-recommended iron deficiency assessment in primary care.

At the same time, these findings highlight the need to investigate whether intravenous iron therapy improves functional capacity and reduces hospitalisations in primary care heart failure populations, as has been demonstrated in hospital-based cohorts. Most randomized trials to date have been conducted in hospital-based heart failure cohorts, with limited representation of older patients typically managed in general practice. Prospective studies designed within primary care, evaluating both the feasibility and clinical effects of intravenous iron supplementation in this population, are therefore warranted.

### Strengths

A major strength of this study is that it represents, to our knowledge, the first investigation of iron deficiency prevalence among patients with heart failure in a primary care setting. The study population reflects real-world clinical practice, with patients recruited from both public and private centres in urban and rural areas, and data were systematically collected from electronic medical records, including laboratory and echocardiographic parameters. Furthermore, iron deficiency was defined using TSAT < 20%, representing a TSAT-based definition rather than the conventional ESC guideline definition of ferritin < 100 µg/L or ferritin 100–299 µg/L with TSAT < 20%. Although current ESC guidelines incorporate ferritin-based criteria, increasing evidence suggests that TSAT may better reflect biologically relevant iron deficiency and identify patients most likely to benefit from iron supplementation [6, 16].

### Limitations

A limitation of this study is the exclusion of patients with missing NYHA class or TSAT values, which may have introduced selection bias. However, included and excluded patients were broadly similar with respect to age, sex, iron status, haemoglobin concentration, and prevalence of anaemia, although included patients had somewhat higher NT-proBNP concentrations. Symptom burden was assessed using NYHA functional class, which is subjective and may be subject to inter-observer variability. Objective measures of functional capacity or patient-reported outcomes were not available. In addition, the study did not assess long-term outcomes such as hospitalisation or mortality, nor did it evaluate the effects of intravenous iron treatment, limiting conclusions regarding clinical benefit.

Due to the cross-sectional design, causality cannot be inferred. Iron deficiency may contribute to symptom burden, but it is also possible that more advanced heart failure contributes to iron deficiency through inflammation, malnutrition or disease severity. Furthermore, inflammatory markers such as C-reactive protein and interleukin-6 were not available. Consequently, residual confounding related to chronic inflammation cannot be excluded.

## Conclusions

In conclusion, iron deficiency is common among patients with heart failure in primary care and is associated with greater symptom burden. These findings support consideration of systematic assessment of iron status in primary care. Future studies are needed to evaluate whether intravenous iron therapy provides similar symptomatic and prognostic benefits in primary care populations as previously demonstrated in hospital-based cohorts.

## Supplementary Information

Supplementary Material 1.

## Data Availability

The datasets generated and analysed during the current study are not publicly available due to ethical and legal restrictions related to patient confidentiality but are available from the corresponding author on reasonable request.

## Abbreviations

NYHA: New York Heart Association
LVEF: Left ventricular ejection fraction
TSAT: Transferrin saturation
TIBC: Total iron-binding capacity
NT-proBNP: N-terminal pro–B-type natriuretic peptide
GDMT: Guideline-directed medical therapy
PHCC: Primary health care centre
