# Supplementary material for: Iron deficiency in primary care patients with heart failure: a cross-sectional study of the heart failure in Southern Sweden (HISS) cohort
Source: BMC Cardiovasc Disord. 2026 Jul 3;26:562. doi: 10.1186/s12872-026-06207-8 (PMC13332607; doi:10.1186/s12872-026-06207-8)
Supplement: Supplementary file 1 — Supplementary Material 1. [file 12872_2026_6207_MOESM1_ESM.docx]

**Table S1. Baseline characteristics of included and excluded patients**

| **Variable** | **Included (n=466)** | **Excluded (n=121)** | **p-value** |
| --- | --- | --- | --- |
| Age (years) | 79 (74-84) | 79 (72-85) | 0.105 |
| BMI (kg/m^2^) | 27.5 (24.9–31.8) | 28.5 (23.9–33.2) | 0.916 |
| Ferritin (µg/L) | 85 (47–164) | 91 (45–164) | 0.632 |
| Transferrin saturation (%) | 25 (19–32) | 23 (17–33) | 0.224 |
| NT-proBNP (ng/L) | 1090 (427–2193) | 824 (275–1919) | 0.023 |
| Haemoglobin (g/L) | 138.0 (16.1) | 138.0 (15.7) | 0.995 |
| Women, n (%) | 191 (41.0) | 56 (46.3) | 0.293 |
| Anaemia, n (%) | 91 (19.6) | 24 (19.8) | 0.940 |

Values are presented as median (IQR) unless otherwise specified. Haemoglobin is presented as mean (SD). Anaemia data missing for one participant.

Abbreviations: BMI, body mass index; NT-proBNP, N-terminal pro-B-type natriuretic peptide; IQR, interquartile range; SD, standard deviation

**Table S2. Agreement between TSAT-based and ESC guideline-based definitions of iron deficiency.**

|  | ESC no ID | ESC ID | **Total** |
| --- | --- | --- | --- |
| TSAT no ID | 172 | 170 | 342 |
| TSAT ID | 1 | 123 | 124 |
| **Total** | 173 | 293 | 466 |

Values are presented as number of patients. Iron deficiency according to the TSAT-based definition was defined as TSAT <20%. Iron deficiency according to the ESC guideline definition was defined as ferritin <100 µg/L, or ferritin 100–299 µg/L with TSAT <20%. Agreement between definitions was low (Cohen’s κ = 0.20, p < 0.001).

**Abbreviations:** ESC, European Society of Cardiology; ID, iron deficiency; TSAT, transferrin saturation.

**Table S3. Baseline heart failure pharmacotherapy according to iron deficiency status**

| **Variable** | **ID n = 124** | **Non-ID**  **n=342** | **p-value** |
| --- | --- | --- | --- |
| RASi, n (%) | 107 (86.3) | 297 (86.8) | 0.877 |
| Beta-blocker, n (%) | 104 (83.9) | 302 (88.3) | 0.207 |
| MRA, n (%) | 51 (41.1) | 113 (33.0) | 0.106 |
| SGLT2 inhibitor, n (%) | 42 (33.9) | 75 (21.9) | 0.009 |
| Loop diuretic, n (%) | 93 (75.0) | 216 (63.2) | 0.017 |
| Intravenous iron treatment during the previous year, n (%) | 6 (4.8) | 7 (2.0) | 0.106 |
|  |  |  |  |

Values are presented as n (%). Group differences were analysed using the χ^2^ test. RASi includes angiotensin-converting enzyme inhibitors, angiotensin receptor blockers, and angiotensin receptor-neprilysin inhibitors.

**Table S4. Sensitivity analysis of the association between iron deficiency and NYHA III–IV using TSAT-based and ESC guideline-based definitions of iron deficiency**

| **Variable** | **Crude OR (95% CI)** | **p-value** | **Adjusted OR (95% CI)** | **p-value** |
| --- | --- | --- | --- | --- |
| Iron deficiency (ESC) | 1.31 (0.83–2.06) | 0.248 | 1.34 (0.81–2.22) | 0.258 |
| Iron deficiency (TSAT) | 2.39 (1.51–3.77) | <0.001 | 1.97 (1.18–3.31) | 0.010 |

Crude and adjusted logistic regression analyses examining the association between iron deficiency and higher symptom burden (NYHA III–IV) using two alternative definitions of iron deficiency. The multivariable model included 428 participants with complete data and comprised age, sex, body mass index, haemoglobin, log NT-proBNP, LVEF <50% (vs ≥50%) and iron deficiency. ESC-defined iron deficiency was defined as ferritin <100 µg/L, or ferritin 100–299 µg/L with TSAT <20%.

**Abbreviations:** CI, confidence interval; OR, odds ratio.

**Table S5. Baseline characteristics by iron deficiency status in patients with LVEF <50%**

| **Variable** | **All (n=270)** | **ID (*n=68*, 25.2%)** | **Non-ID (*n=202, 74.8%*)** | **p-value** |
| --- | --- | --- | --- | --- |
| Age (years) | 79 (72–83) | 79 (73–84) | 79 (72–83) | 0.556 |
| BMI (kg/m²) | 27.2 (24.7–31.8) | 27.2 (24.1–33.0) | 27.2 (24.8–31.3) | 0.762 |
| Ferritin (µg/L) | 88 (49–167) | 53 (30–102) | 101 (58–187) | <0.001 |
| Transferrin Saturation (%) | 25 (20–32) | 16 (13–19) | 28 (23–34) | <0.001 |
| NT-proBNP (ng/L) | 1183 (418–2482) | 1433 (448–3490) | 1052 (412–2210) | 0.170 |
| Haemoglobin (g/L) | 138.5 (16.0) | 133.5 (15.8) | 140.2 (15.7) | 0.003 |
| Women, n (%) | 102 (37.8) | 31 (45.6) | 71 (35.1) | 0.125 |
| Anaemia, n (%) | 55 (20.4) | 21 (30.9) | 34 (16.8) | 0.013 |

Values are presented as median (IQR) unless otherwise specified. Haemoglobin is presented as mean (SD). Anaemia data missing for one participant.

Abbreviations: BMI, body mass index; ID, iron deficiency; NT-proBNP, N-terminal pro-B-type natriuretic peptide; IQR, interquartile range; SD, standard deviation

Iron deficiency was defined as transferrin saturation (TSAT) <20%.
